# A Value Framework for Evaluating Population Genomic Programs: A Mixed Methods Approach †

**DOI:** 10.3390/jpm15070307

**Published:** 2025-07-12

**Authors:** David Campbell, Scott Spencer, Ashley Kang, Rajshree Pandey, Sarah Katsandres, David Veenstra

**Affiliations:** 1 Curta Inc., Seattle, WA 98104, USA; david.campbell@curta.com (D.C.); ashley.kang@curta.com (A.K.); rajshree.pandey@curta.com (R.P.); sarah.katsandres@curta.com (S.K.); david.veenstra@curta.com (D.V.); 2 Illumina Inc., San Diego, CA 92122, USA; 3 The CHOICE Institute, Department of Pharmacy, University of Washington, Seattle, WA 98195, USA

**Keywords:** precision medicine, population health genomics, genome sequencing, population sequencing, healthcare infrastructure, genomic medicine

## Abstract

**Background/Objectives**: Value frameworks are useful tools to explicitly define the dimensions and criteria important for decision-making, but no existing frameworks capture the broad value domains of population genomic programs. Using a mixed methods approach, we aimed to develop a novel value framework for evaluating population genomic programs (PGPs). **Methods**: We first conducted a targeted literature review of published evidence on the value of PGPs and existing frameworks to evaluate and quantify their impact. Value domains and elements were extracted and summarized to develop a preliminary framework. Semi-structured stakeholder interviews on the preliminary framework were conducted from March 2024 to October 2024 with 11 experts representing 9 countries. A thematic analysis of interview transcripts was conducted to map value elements to domains of the final framework. **Results**: We identified 348 potentially relevant articles from MEDLINE-indexed and the gray literature sources. After title and abstract screening, 23 articles met the inclusion criteria and underwent full-text review, and 8 reported value elements were extracted and mapped to a preliminary framework for testing in interviews. Stakeholder themes were summarized into the value domains and elements of the final framework, which included health as a primary domain, education and research, enterprise and finance, and labor as the core domains, and agriculture and security as extended domains. Domains and elements may be excluded based on stakeholder objectives and program characteristics. **Conclusions**: This novel framework for assessing the comprehensive value of PGPs provides a foundational step to assess the value of these programs and may promote more efficient and informed allocation of resources.

## 1. Introduction

There has been significant interest across many countries to implement genomic programs particularly related to the broad-reaching impacts of genomics on health, such as precision medicine, disease surveillance, and diagnostics, as well as areas outside of health such as agriculture and national security [1,2,3,4,5,6]. Successful implementation requires investment in scientific expertise, sequencing capabilities, and bioinformatics, generally at scale, to benefit the broad population within a given country. Population genomics programs (PGPs) are often positioned as a key step in achieving these goals as a result of various research and/or clinical activities. A population genomics program can be defined broadly as a large, national, or multi-national comprehensive initiative that aims to capture, analyze, and leverage genetic information for the benefit of a population.

A challenge for decision-makers is understanding the potential far-reaching benefits of a PGP [7,8,9]. Population genomic programs may require substantial upfront and continued investment, with a years-long journey from program design, through implementation, and translation [10]. In addition, PGPs uniquely require the secure handling of massive datasets, substantial infrastructure to identify and implement positive use cases into clinical practice, and ethical oversight for the appropriate use of individual data. To support informed decisions on investment in PGPs there is a need to comprehensively capture and describe the impacts of these programs beyond the individual, which might include the family, caregivers, the wider healthcare system, and society. The impacts and benefits of PGPs may take years or even decades to fully materialize especially as they relate to areas like research and development. Challenges to fully capturing the value and benefits of these programs which are complicated by the breadth and timing of impacts, introduce the risk of underinvestment in these programs and the potential for the misallocation of resources [11,12].

Value frameworks are common and useful tools to explicitly define the dimensions and criteria considered integral to decision-makers. There are many value frameworks within healthcare covering different scopes and intended applications [12,13,14]. While frameworks have been developed for specific genetic tests [15,16,17,18,19], no existing frameworks have been developed to capture the value domains and applications of PGPs. The goal of this study is to develop a novel value framework for evaluating population genomic programs using a mixed methods approach that included both a review of published literature and interviews with key stakeholders.

## 2. Materials and Methods

We first conducted a targeted literature review to synthesize the published evidence on the value of population genomic programs and existing frameworks used to evaluate and quantify their impact. Searches were focused on studies published from January 2013 to December 2023 in the MEDLINE database (Table S1). A manual search for published reports and white papers not indexed in MEDLINE was also performed.

Studies that examined genomics programs implemented at a national or regional level for either screening and diagnostic purposes that reported economic value outcomes (i.e., indirect, direct, or induced) were included. Studies that did not involve a large, publicly funded genomic program or only reported clinical outcomes were excluded. Editorials, opinion pieces, narrative reviews, study protocols, and conference abstracts were excluded, as were non-English language studies (Table S2). Identified value domains and value elements of these programs were extracted and summarized for the development of a stakeholder interview discussion guide (Table S3).

Studies were screened for inclusion and exclusion criteria by an independent investigator using Nested Knowledge software (Nested Knowledge, Saint Paul, MN, USA) [20]. Data were extracted by one reviewer and validated by an independent reviewer.

We next conducted semi-structured stakeholder interviews to gather current perspectives on the value domains and elements of population genomic programs initially identified through the literature search. A purposeful sampling technique was employed to identify 11 expert stakeholders with relevant expertise in the development or evaluation of population genomic programs, health economics, or value assessment. Expertise was assessed by peer-reviewed publications, presentations, the role supporting the development, implementation, or the evaluation of population genomic programs. No individually identifiable health information was collected, and the study was exempt from institutional review board review.

We conducted online interviews from March 2024 to October 2024 with 11 experts across North America, South America, Europe, Asia, and Australia providing diverse perspectives across variable geographies, populations, and local health systems. During the interviews, stakeholders were presented with findings from the literature review on the value of PGPS and preliminary value framework and asked to provided perspectives on (1) completeness of literature review, (2) preferred approach to value assessment, (3) impact of objectives on value assessment and measurement, (4) priority and relevancy of value domains and elements, and (5) country and healthcare system-specific considerations. To limit bias and promote consistency across interviews, a single internal investigator conducted all the interviews, and a second investigator captured notes during the interviews.

We conducted a thematic analysis of the stakeholder interview transcripts to identify the most cited value domains of population genomic programs [21]. Stakeholder recommendations for value elements were refined for clarity, aligned to common groups, and mapped under these domains to create a comprehensive value framework for population genomic programs that reflects global stakeholder perspectives.

## 3. Results

**Figure 1:**
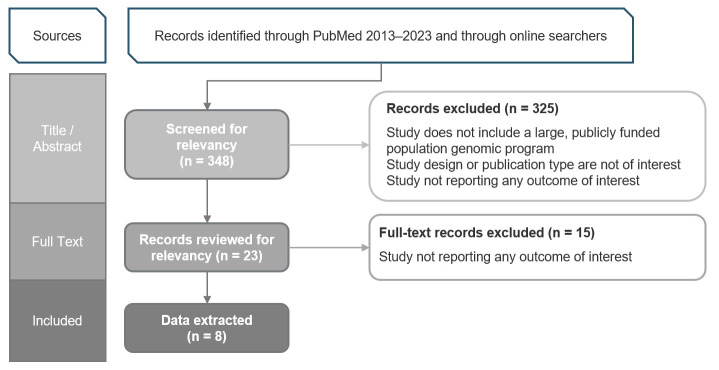
Results of population genomic program literature review.

We identified 348 potentially relevant articles for screening from MEDLINE-indexed and gray literature sources. After title and abstract screening, 23 articles met the inclusion criteria and underwent full-text review. Among articles that underwent full-text review, eight reported outcomes of interest and underwent data extraction (Figure 1).

Studies meeting inclusion criteria represented diverse populations with varied levels of healthcare system nationalization. The majority of included articles examined the value of population genomics in countries with established programs, including the United Kingdom (3), the United States (2), and Australia (1) [22,23,24,25,26,27]. The remaining two articles examined the value of PGPs from a global perspective [28,29]. Characteristics of the included articles are presented in Table 1. All articles included benefits to health outcomes as a central value element of PGPs, as well as cited applications to promote health, included screening, diagnosis, pharmacogenomics, personalized treatment, and management. The value of genomics to education and research was also cited in all articles and included benefits to improving research and development. Several articles referenced the direct and indirect value of PGPs through company creation, investment, employment, and productivity through a healthier workforce. More than half of the articles (63%) included one or more financial outcomes as a benefit to PGPs. Non-human applications of genomics in agriculture, the environment and national security were less cited areas of value. Two articles highlighted the role of genomics as part of public health strategies for the COVID-19 pandemic.

A recent report commissioned by the American Society of Human Genetics (ASHG), quantified the economic impact of human genetics and genomics in the United States including direct, indirect, and induced effects [26]. Using an IMPAN input-output model framework for the analysis, the study estimated $265 billion in total economic impact in the U.S. from the human genomics and genetics sector. Overall, the analysis estimates a return on investment of $4.75 for every dollar of federal funds invested in genomics. While limited to only human genetics and genomics only, the study highlights the substantial value from genomic programs from direct, indirect, and induced effects.

Across studies, six value domains were identified: health, education, finance, labor, agriculture, and defense. No single article identified in the literature review included all six value domains (Table 1).

**Figure 2:**
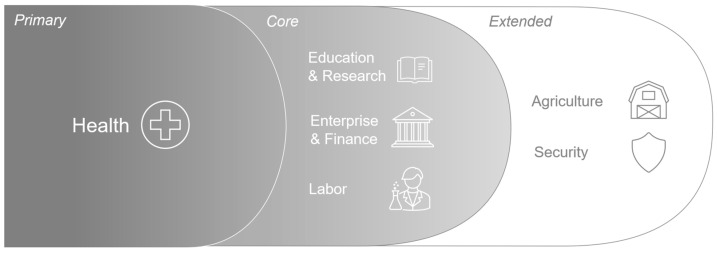
Value domains of population genomic programs.

The experts were recruited from nine countries which included Brazil, Denmark, England, France, Singapore, Sweden, Switzerland, Thailand, and the United States. These stakeholders included eight experts actively involved in the development or evaluation of PGPs, two health economists, and one expert in health technology assessment (HTA) and value assessment. Stakeholders refined the preliminary value domains to six value domains (Figure 2). As the design of PGPs varies in the real world, the value domains were designed to capture broad potential value across the spectrum of PGPs with the flexibility for users to only include relevant value domains and elements.

Stakeholders universally agreed that health is the primary source of value for population genomic programs, and these programs also importantly create value that extends beyond health to other domains. In addition to diagnostic services, personalized medicine, and rational drug development, stakeholders identified public health strategy development and screening programs in pediatric, rare disease, high-risk, general, and newborn populations as valuable applications within the domain of health.

Education was viewed as a core area of value to genomic programs. However, stakeholders shared that depending on the governmental structure these applications may be better categorized under the research domain. Within this area, value may be realized from the development of biobank research databases. The establishment of genomic research programs may strengthen universities, create new education and training opportunities, and support the generation of new knowledge, publications, and intellectual property.

A similar level of priority was reported for the domains of finance and labor as PGPs may stimulate economic development through job creation, investment, and tax revenues. Stakeholders noted benefits to enterprise, innovation, technology, and sector leadership as important value drivers of genomic programs for policy-makers. Genomic programs may accelerate the growth of high-skill, science, and technology jobs and the retention of educated labor in the region. Workforce participation and output can also increase through improved health of labor from the clinical impacts of PGPs.

Beyond these areas, the value of population genomic programs may extend to agriculture and security. Agriculture benefits have been demonstrated in the optimization of livestock and crops through enhanced yield and promotion of desired attributes. Genomic programs aimed at reducing the need for water and pesticides can support environmental conservation efforts. Initially characterized in the preliminary framework as defense, stakeholders reported the domain of security better captured the perceived value and application of population genomic programs to support public health strategy and preparedness. Investment in these programs provides the knowledge, infrastructure, and capacity to incorporate genomics into national security and risk mitigation strategies.

Stakeholders’ ratings of the level of priority of each value domain of the preliminary framework were aggregated to create primary, core, and expanded value domains. Value elements were extracted and summarized from interview transcripts, and a thematic analysis was performed to align value elements of similar characteristics or impacts together. The consolidated inventory of value elements were placed under stakeholder-informed value domains to construct the final value framework (Table 2).

## 4. Discussion

Genomics and its integration into health systems around the world have made tremendous advancements over the past three decades. This has led to increased and continued interest in the creation and funding of these programs across the globe. However, prior value assessments of population genomic programs have failed to capture the holistic value of these programs beyond human applications, which presents challenges when countries and decision-makers consider whether to invest in PGPs. Informed by the literature and expert stakeholders, we developed a novel value framework to comprehensively describe the value of population genomic programs.

The value framework we developed includes the following value domains: (1) health, (2) education and research, (3) enterprise and finance, (4) labor, (5) agriculture, and (6) security. The value domains of the framework cover broad areas to capture the wide and expanding list of PGP applications. Importantly, the framework was designed to be flexible by allowing users to consider only the domains and applications relevant to the genomic program being assessed. This broad set of considerations aligns with a program’s unique objectives and design. Stakeholders emphasized that any framework should be adaptable to accommodate the range of population genomic program designs, the stage of program implementation, healthcare systems, and the political structure of the population. The comprehensive and adaptable structure of this novel framework can help policy and decision-makers better understand the value of these programs, and support more informed decision-making. The flexible approach is particularly useful at the early stages of designing a PGP when different program structures are under consideration. Applying this framework, genomic programs which include the establishment of research centers may create value across more domains, compared to programs limited in scope to clinical application of genomic information only. The benefits from additional value domains and elements can be weighed against PGP design choices with greater resource needs.

A previous systematic review of existing value frameworks for health technologies reported value criteria for next-generation sequencing and comprehensive genomic profiling may serve in a complimentary role to our framework [12]. First, our value framework can establish the broad value domains and elements relevant to a PGP and the value assessment stakeholders, which subsequently could apply the comprehensive list of value criteria by Augustovski et al. Similarly to our value framework, value criteria included clinical and safety health impacts, economic, environmental, and broader social impacts to industrial promotion, job creation and technology transfer. In addition, equity, ethical and legal aspects, quality assurance, and data governance were identified as value criteria for genomic sequencing. These additional value criteria were frequently cited in the literature and were raised in the stakeholder interviews as important considerations for the assessment and implementation of PGPs. In addition, proper data systems to secure and protect data are viewed as essential components to any population genomic program. Considering the significant duration of time from initial PGP investment to achieved outcomes, uncertainty in the realization of long-term impacts exists for all value elements. Previous research has advocated for the inclusion of value of knowing (value from reducing uncertainty) and insurance value (value from protection from financial health risk) in the value assessment of personalized medicine and precision medicine [30]. PGPs may generate significant utility in value of knowing and value of insurance domains by informing patients, clinicians, and health systems on the presence or risk for clinical conditions.

Due to the ability for genomic and personalized programs to create large-scale impacts on morbidity and mortality, there is great potential for PGPs to widen or to close existing disparities in health outcomes [31,32]. Therefore, health equity and a holistic view of PGP impacts need to be considered in the establishment and assessment of these programs. A recent international workshop explored the issue of the value of large-scale genomic programs for society [33]. Similarly to our study, workshop participants highlighted the broader societal implications of these programs that extend beyond direct health benefits. The workshop also generated specific planning and implementation recommendations to optimize PGP outcomes. The workshop’s primary recommendation for open discourse on the practical scope and limitations of genomic medicine may benefit from the use of our framework. Additional recommendations included the need for additional evidence to be generated on the economic value of PGPs, consideration of the diverse patient, family and physician experiences and perceptions, and balancing personalized medicine with population health goals. The failure to appropriately capture the value of PGPs may worsen equity issues by inhibiting investment to expand or create new genomic programs for populations that have been historically underserved. The use of this comprehensive value framework may help address equity issues by promoting investment in genomic sequencing of subgroups that have been historically underrepresented in genomic research. However, further research is needed to assess the long-term impacts of PGP design on health equity and the application of value frameworks within multi-stakeholder efforts to reduce existing inequalities.

The process to develop the novel framework described in this article has strengths and limitations. One limitation is the qualitative nature of mapping value applications from the literature review to value domains, which involved interpretation and subjectivity by the research team. The semi-structured interviews only included 11 experts across nine countries. The views from these stakeholders may not be widely generalizable. The framework does not include all possible applications or sources of value for population genomic programs, but only those cited most frequently. The framework developed in this study does not provide guidance for the quantification of value or summation of value across applications and domains. As with other health technology assessments, the value judgment of population genomic programs will be impacted by the stakeholder perspective and should be tailored to reflect unique societal priorities and values. Further research is needed on the measurement of value within the identified applications and value domains of the framework. The framework was not designed to address the impact of PGP implementation on realized outcomes. Implementation of PGPs is particularly challenging due to the significant scale of infrastructure, data collection, analysis, and coordination between research and clinical application that is required. There are also important ethical and legal considerations related to the collection and use of genomic data and the appropriate mechanisms to safeguard patients [34,35,36,37]. The implementation of proper data security and maintaining ethical standards may impact the realized value of any PGP [36]. Further research on the impact of implementation of PGPs may improve the utility and precision of the value framework for more informed decisions.

## 5. Conclusions

The stakeholder-informed framework developed in this study establishes the foundation for evaluating PGPs by identifying the value domains and applications relevant to specific stakeholder priorities. The application of this comprehensive value framework may advance population genomic programs by providing a platform for consistent and appropriate assessments informing PGP investment decisions.

## Figures and Tables

**Table 1:** Study characteristics and value domains of literature review.

Author	Battelle	Deloitte	Livesey	WEF	InGeNA	ASHG	BIA	PPP
Year	2013	2015	2019	2020	2021	2021	2022	2022
Type	Model	Review	Model	Review	Review	Model	Review	Review
Geography	U.S.	U.K.	U.K.	Global	AUS	U.S.	U.K.	Global
Value Domains								
Health	x	x	x	x	x	x	x	x
Education	x	x	x	x	x	x	x	x
Finance	x	x			x	x	x	
Labor	x	x			x	x	x	x
Agriculture						x		x
Defense							x	x

Abbreviations: ASHG = American Society of Human Genetics; AUS = Australia, BIA = UK BioIndustry Association; PPP = Public policy projects; U.K. = United Kingdom; U.S. = United States; WEF = World Economic Forum.

**Table 2:** Value framework for population genomic programs.

Value Domains		Value Elements
Primary	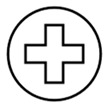 Health	o Personalized medicine; o Gene editing and therapy; o Rational drug development; o Diagnostic services; o Public health strategy development and screening: - Pediatric/rare disease; - High-risk population; - General population; - Newborn population.
Core	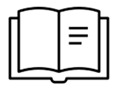 Education & Research	o Biobank research database; o Education and training programs; o Establish centers of excellence; o Minable big data; o Patents-intellectual property; o Publication opportunities; o University funding.
	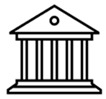 Enterprise & Finance	o Drug development; o Pharma investment; o R&D commercialization returns; o Tax revenue; o Vaccine development; o Venture investment.
	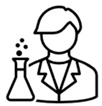 Labor	o Growth in high-skill, science, and technology jobs; o Retention and expansion of educated work force; o New opportunities for under-represented populations; o Healthier and more productive workforce.
Extended	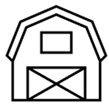 Agriculture	o Agriculture optimization: - Promote desired livestock and crop attributes; - Enhance growth/yield; - Resist pests and disease. o Environmental conservation: - Reduce water consumption; - Antimicrobial stewardship.
	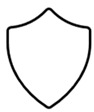 Security	o Public health strategy and preparedness; o Protect and optimize health; o Critical security technology; o Surveillance (pathogen tracking, environmental exposures); o Risk mitigation for economic resiliency.

## Data Availability

The data underlying this article will be shared on reasonable request to the corresponding author.

## Supplementary Materials

The following supporting information can be downloaded at: https://www.mdpi.com/article/10.3390/jpm15070307/s1, Table S1: Literature review MEDLINE search; Table S2: Study eligibility criteria for targeted review; Table S3: Value elements of genomic programs reported in the literature. Table S4: PGP Value Review and Conceptional Framework Interview Guide.
