# Supplementary material for: A Value Framework for Evaluating Population Genomic Programs: A Mixed Methods Approach
Source: J Pers Med. 2025 Jul 12;15(7):307. doi: 10.3390/jpm15070307 (PMC12296045; doi:10.3390/jpm15070307)
Supplement: Supplementary file 1 [file jpm-15-00307-s001.zip › jpm-3716382-supplementary.pdf]

Table S1. Literature review MEDLINE search.

S#	Search Query	Search Hits
1	("Genomics"[Mesh] OR "Genomics"[MAJR] OR "genomics"[tiab]) OR "Genetic Profile"[Mesh] OR "Genetic Diseases, Inborn/diagnosis"[MAJR] OR (genetic AND (service or counsel* OR screening?)) OR (genomic? AND (resource OR service? or portfolio)) OR (Prenatal AND (Noninvasive OR Screening)) AND (("mass" OR "carrier") AND "screening") OR "Molecular Diagnostic Techniques"[Majr] OR (("molecular" OR "genetic") AND "techni*") OR ("national" AND "genomic*") OR "Genomics"[MAJR] OR "genomic screening"[tiab:~0] OR "genomics program"[tiab:~1] OR "genomics programme"[tiab:~1] OR "NGP"[tiab] OR "diagnostic genomics"[tiab:~0]	785,383
2	"Genetics, Medical/economics"[MeSH] OR "Genomics/economics"[MAJR] OR "Health Services Research/economics"[MAJR] OR "Public Health"[MAJR] OR "Pharmacogenetics/economics"[MeSH] OR "Human Genome Project"[MeSH] OR "Genetic Testing/economics"[MeSH]	2,084,904
3	("Health Plan Implementation"[Mesh] OR "Translational Research, Biomedical"[Mesh]) OR (implement* OR disseminat* OR translation OR transfer OR incorporation OR integration)	3,960,502
4	((((((("Health Resources"[Mesh]) OR "National Health Programs"[Mesh]) OR "Health Systems Plans"[Mesh]) OR "Program Development"[Mesh]) OR "Health Policy"[Mesh]) OR "Delivery of Health Care"[Mesh]) OR "Health Services Accessibility"[Mesh]) OR ("Community Health Services/organization and administration"[Mesh] OR "Community Health Services/standards"[Mesh]) OR "health system"[tiab:~1]	1,459,566
5	(#1 AND #2) AND #3 AND #4	621
6	#5 NOT (comment[pt] OR editorial[pt] OR letter[pt] OR "case reports"[pt])	602
7	#6 NOT (animals[MeSH] NOT humans[MeSH])	601
8	Filters: from 2013 – 2023	337
9	Filter: English language	**322**

Table S2. Study eligibility criteria for targeted review.

Category	Inclusion Criteria	Exclusion Criteria
Disease or clinical indication	Any	NA
Population	Any	NA
Intervention and Comparators	National genomics programs and pilots implemented at a national or regional level for both, screening and diagnostic purposes	Programs related to therapeutic genomic programs and not implemented at the national level
Geography	Any	NA
Study Designs and Publication Types	Peer-reviewed articles where genomic programs are described<br>White papers and government documents	Editorials, opinion piece articles, narrative (non-systematic) reviews,<br>Study protocols<br>Conference abstracts

Outcomes	Economic value outcomes:<br>Indirect<br>Direct<br>Induced	Reporting only clinical outcomes
Language	English language	Not published in English language
Date of publication	2013 to 2023	NA

Table S3. Value elements of genomic programs reported in literature.

Publication	Reported Value Elements and Applications of Genomic Programs
The Impact of Genomics on the U.S. Economy [25]	Direct and indirect employment<br>Health benefits<br>Investment revenue<br>Research and development
Genomics in the UK: An Industry Study for the Office of Life Sciences [22]	Clinical services<br>Diagnostics<br>Drug development<br>Employment<br>New company formation<br>Research and publications
Modeling the Possible Returns to the NHS from Private Sector Use of the 10k Genomes Database [23]	Data licensing<br>Drug development<br>Pharmacogenomics<br>Screening and diagnosis
Global Data Access for Solving Rare Disease: A Health Economics Value Framework [28]	Clinical treatment and management<br>Clinical trial / research and development<br>Diagnostic benefits<br>Value of information
Valuing the Impact of Genomics on Healthcare in Australia [27]	Diagnosis<br>Improved health outcomes / efficiency<br>National reputation and strength of industry<br>Personal utility<br>Research and development<br>Screening<br>Treatment<br>Workforce participation
The Economic Impact and Functional Applications of Human Genetics and Genomics [26]	Diagnosing diseases and disorders<br>Direct and indirect jobs supported<br>Environmental genomics and metagenomics<br>Gene editing and gene therapy<br>Human-microbe interaction<br>Identifying predisposition to disease and disorders<br>Minable big data / discovery science<br>Pharmacogenomics<br>Rational drug development<br>Tax revenue
Genomics Nation 2022: Highlighting Future Opportunities for the UK Genomics Sector [24]	Company creation and growth<br>Pandemic response<br>Pharmacogenomics<br>Public investment

	Rational drug development<br>Venture investment
Bringing the Benefits of Genome Sequencing to the World [29]	Agriculture<br>Drug and vaccine development<br>Education and talent development<br>Environment<br>Genetic testing and diagnostics<br>Health crisis management<br>Intellectual property<br>Job creation<br>Personalized medicine<br>Research data sets<br>Technology and innovation

Table S4. PGP Value Review and Conceptional Framework Interview Guide.

Number	Question
1.	Can you please share a brief background about yourself and work as it relates to population genomic program development or value assessment.
2.	Have you seen any value assessment frameworks for PGPs that you think are sufficiently comprehensive?
3.	What are your initial thoughts on how countries should approach value assessment of population genomic programs?
4.	How do program objectives impact views on value assessment / measurement?
5.	PGPs may impact many departments of government responsibility. Do the six listed here (health, education, finance, labor, agriculture, defense) cover all the relevant domains? If no, what is missing?
6.	How do you view the level of priority and relevancy of these domains?
7.	How do the relevant domains change if the PGP is focused on research vs clinical implementation?
8.	Are there other value elements that should be captured within a PGP value framework for each domain (health, education, finance, labor, agriculture, defense)?
9.	What outputs do you think are most important for the design of a PGP value framework?
10.	Is there any additional information, not covered earlier that is important to consider for the development of a population genomic program value framework?
